# Comparative Efficacy and Safety of Angiotensin-Converting Enzyme Inhibitors, Angiotensin Receptor Blockers, and Calcium Channel Blockers in Hypertensive Patients With Chronic Kidney Disease: A Systematic Review

**DOI:** 10.7759/cureus.78845

**Published:** 2025-02-11

**Authors:** Mouzafar A Mhmndar, Taranpreet Singh, Iram Fatima, Abdullah Shehryar, Moosa R Zaidi, Muhammad Mairaj, Safiyyah M Khan, Nikhil Deep Kolanu, Saba Ahmed, Anosha Kamal, Abdur Rehman, Maria Quinn

**Affiliations:** 1 Health, Dubai Academic Health Corporation, Dubai, ARE; 2 Internal Medicine, Mahatma Gandhi Mission's (MGM) Medical College and Hospital, Navi Mumbai, IND; 3 Internal Medicine, Holy Name Medical Center, Teaneck, USA; 4 Internal Medicine, Allama Iqbal Medical College, Lahore, PAK; 5 Acute Medicine, North Middlesex University Hospital, London, GBR; 6 Medicine and Surgery, Chandka Medical College, Larkana, PAK; 7 Internal Medicine, Basaveshwara Medical College, Chitradurga, IND; 8 Internal Medicine, China Medical Univesity, Shenyang, CHN; 9 Anesthesia and Critical Care, Health Service Executive, Kerry, IRL; 10 Cardiology, Tabba Heart Institute, Karachi, PAK; 11 Cardiology, Dow University of Health Sciences, Karachi, PAK; 12 Surgery, Mayo Hospital, Lahore, PAK; 13 Internal Medicine, Jinnah Hospital Lahore, Lahore, PAK

**Keywords:** ace inhibitors, angiotensin receptor blockers, antihypertensive therapy, calcium channel blockers, cardiovascular outcomes, chronic kidney disease, egfr, hypertension, proteinuria, systematic review

## Abstract

This systematic review evaluates the comparative efficacy and safety of antihypertensive therapies, including angiotensin-converting enzyme inhibitors (ACEis), angiotensin receptor blockers (ARBs), and calcium channel blockers (CCBs), in managing hypertension among chronic kidney disease (CKD) patients. A comprehensive search adhering to Preferred Reporting Items for Systematic Reviews and Meta-Analyses (PRISMA) guidelines was conducted across five databases, identifying eight randomized controlled trials (RCTs) published in the last five years. The included studies examined diverse populations, ranging from pediatric to elderly CKD patients, with interventions tailored to specific subgroups, including those with proteinuria or diabetic kidney disease. Key outcomes assessed included changes in estimated glomerular filtration rate (eGFR), proteinuria reduction, cardiovascular events, and treatment-related adverse effects. Findings highlighted the superiority of ACEis and ARBs in reducing proteinuria and slowing CKD progression, particularly in proteinuric patients, while CCBs were effective in blood pressure control and improving cardiovascular parameters. However, no head-to-head trials directly comparing renin-angiotensin-aldosterone system (RAAS) inhibitors (ACEis/ARBs) and CCBs were identified, limiting definitive conclusions regarding their relative efficacy. Limitations such as small sample sizes and short follow-up durations were also noted in some studies. This review underscores the importance of individualized therapy based on patient-specific factors to optimize renal and cardiovascular outcomes. Further research is recommended to explore long-term outcomes and combination therapies in this population.

## Introduction and background

Hypertension is a significant public health concern and a leading risk factor for chronic kidney disease (CKD), a condition characterized by progressive renal function decline that may lead to end-stage renal disease (ESRD) [1]. The coexistence of hypertension and CKD creates a complex interplay where elevated blood pressure exacerbates kidney damage, while impaired renal function aggravates hypertension [2]. This bidirectional relationship underscores the critical need for effective blood pressure management strategies tailored for CKD patients. Antihypertensive medications, particularly angiotensin-converting enzyme inhibitors (ACEis), angiotensin receptor blockers (ARB), and calcium channel blockers (CCB), are widely used to achieve blood pressure targets and mitigate CKD progression and cardiovascular complications [3]. However, there is ongoing debate regarding the comparative long-term efficacy and safety of these drug classes in CKD patients. Understanding the nuances of these treatment options is essential for optimizing patient outcomes, particularly given the global burden of hypertension-related kidney disease.

The renin-angiotensin-aldosterone system (RAAS) plays a pivotal role in the pathophysiology of hypertension and CKD [4]. Both ACEis and ARBs target RAAS to reduce intraglomerular pressure, proteinuria, and inflammation, thereby slowing CKD progression. While ACEis are often recommended as the first-line therapy for hypertensive CKD patients, ARBs provide an alternative for those intolerant to ACEis due to side effects like cough or angioedema [5]. Calcium channel blockers, on the other hand, lower blood pressure by reducing vascular smooth muscle contraction and are particularly beneficial for salt-sensitive hypertension. Despite their widespread use, the relative effectiveness of these agents in preventing long-term complications, such as ESRD and cardiovascular events, remains an area of active investigation [6].

This systematic review is guided by the Population, Intervention, Comparison, Outcome (PICO) framework [7] to ensure a structured and focused approach to evaluating the available evidence. The population includes adult and pediatric patients diagnosed with CKD and hypertension, encompassing individuals at varying stages of CKD, both with and without proteinuria, as well as those undergoing maintenance hemodialysis. The intervention under investigation involves antihypertensive therapy using ACEis, ARBs, or CCB. The comparison focuses on a detailed analysis of these three drug classes, emphasizing their effectiveness in reducing blood pressure, slowing CKD progression, and mitigating cardiovascular risks. Key outcomes assessed in this review include long-term kidney function preservation, such as glomerular filtration rate stability, reduction in proteinuria, incidence of ESRD, cardiovascular event rates, treatment-related adverse effects, and overall mortality.

The primary objective of this systematic review is to identify the optimal therapeutic approach for hypertensive CKD patients by highlighting differences in efficacy, safety, and long-term benefits among ACEis, ARBs, and CCBs. While ACEis have demonstrated superior long-term benefits in terms of renal protection and reduction in all-cause mortality compared to ARBs, the latter are often prescribed more frequently in CKD patients. This is primarily due to the better tolerability profile of ARBs, as ACEis are associated with side effects such as cough and angioedema, which can limit their use in certain patients. Angiotensin receptor blockers provide a valuable alternative for those who are intolerant to ACEis while still offering significant renoprotective effects. By aligning the research design with the PICO framework, this review ensures a robust methodology to address clinically relevant questions and provide actionable insights for healthcare providers involved in the management of CKD patients. This structured approach underscores the importance of evidence-based decision-making in tailoring antihypertensive therapy to improve patient outcomes.

## Review

Materials and methods

Search Strategy

The search strategy for this systematic review was developed in adherence to Preferred Reporting Items for Systematic Reviews and Meta-Analyses (PRISMA) guidelines [8] to ensure a comprehensive and methodologically rigorous approach. Electronic databases, including PubMed, Excerpta Medica database (Embase), Cochrane Central Register of Controlled Trials (CENTRAL), Web of Science, and ClinicalTrials.gov, were searched for randomized controlled trials (RCTs) published within the last five years. The search combined Medical Subject Headings (MeSH) terms and free-text keywords such as “Chronic Kidney Disease,” “ACE inhibitors,” “Angiotensin Receptor Blockers,” “Calcium Channel Blockers,” “Proteinuria,” and “eGFR.” Boolean operators were used to enhance precision. Inclusion criteria focused on hypertensive CKD patients, comparing ACEis, ARBs, and CCBs with long-term renal and cardiovascular outcomes. Articles were screened in two stages, namely, title/abstract screening followed by full-text review, with grey literature sources searched to minimize bias. All studies were managed using reference management tools, ensuring systematic organization and deduplication. This strategy facilitated the identification of high-quality, relevant studies for synthesis and analysis.

Eligibility Criteria

This systematic review included studies that focused on hypertensive patients with CKD receiving antihypertensive therapy with ACEis, ARBs, or CCBs. Eligible studies were required to report on long-term outcomes such as changes in estimated glomerular filtration rate (eGFR), reduction in proteinuria, progression to end-stage kidney disease (ESKD), cardiovascular events, or mortality. Only RCTs or clinical trials published within the last five years were included to ensure the findings reflect the most recent and relevant evidence. Studies involving both adult and pediatric CKD populations were considered, provided they included sufficient detail on interventions, comparators, and measured outcomes. The review was restricted to articles published in English, and trials with incomplete or unclear outcome reporting were excluded [9].

Exclusion criteria included observational studies, reviews, case reports, and editorials, as well as trials focusing on non-hypertensive CKD populations or interventions outside the scope of ACEis, ARBs, or CCBs. Studies with short follow-up durations or those that did not report key outcomes related to CKD progression or cardiovascular health were also excluded. These stringent eligibility criteria ensured the inclusion of high-quality evidence directly addressing the comparative efficacy and safety of these antihypertensive therapies in CKD patients.

Data Extraction

Data extraction for this systematic review was performed using a standardized form to ensure consistency and accuracy. Key details extracted from each included study comprised author names, publication year, population characteristics (e.g., CKD stage, proteinuria status, hypertension severity), intervention details (e.g., ACEis, ARBs, or CCBs, including dosage and duration), comparators, and reported outcomes such as changes in eGFR, proteinuria reduction, progression to ESKD, cardiovascular events, and mortality. Secondary data, such as adverse effects and safety parameters, were also recorded. Two independent reviewers conducted the data extraction process, with discrepancies resolved through discussion or consultation with a third reviewer. This rigorous approach minimized bias and ensured a comprehensive and reliable synthesis of the evidence. All data were organized in tabular format to facilitate subsequent analysis and comparison across studies.

Data Analysis and Synthesis

Data analysis and synthesis in this systematic review were performed through a qualitative narrative approach due to the heterogeneity in study populations, interventions, and outcome measures. The extracted data were grouped and analyzed based on key themes, such as the comparative efficacy of ACEis, ARBs, and CCBs in reducing proteinuria, slowing CKD progression, and improving cardiovascular outcomes. Trends and patterns were identified to highlight the relative benefits and risks of each antihypertensive class across different subpopulations, such as those with proteinuric versus non-proteinuric CKD or diabetic versus non-diabetic patients. A narrative synthesis was employed to integrate findings from individual studies, with special attention to the consistency of results, potential biases, and study limitations. This method provided a comprehensive understanding of the comparative outcomes and informed clinical implications while also identifying gaps in the current evidence base that warrant further research.

Results

Study Selection Process

**Figure 1:**
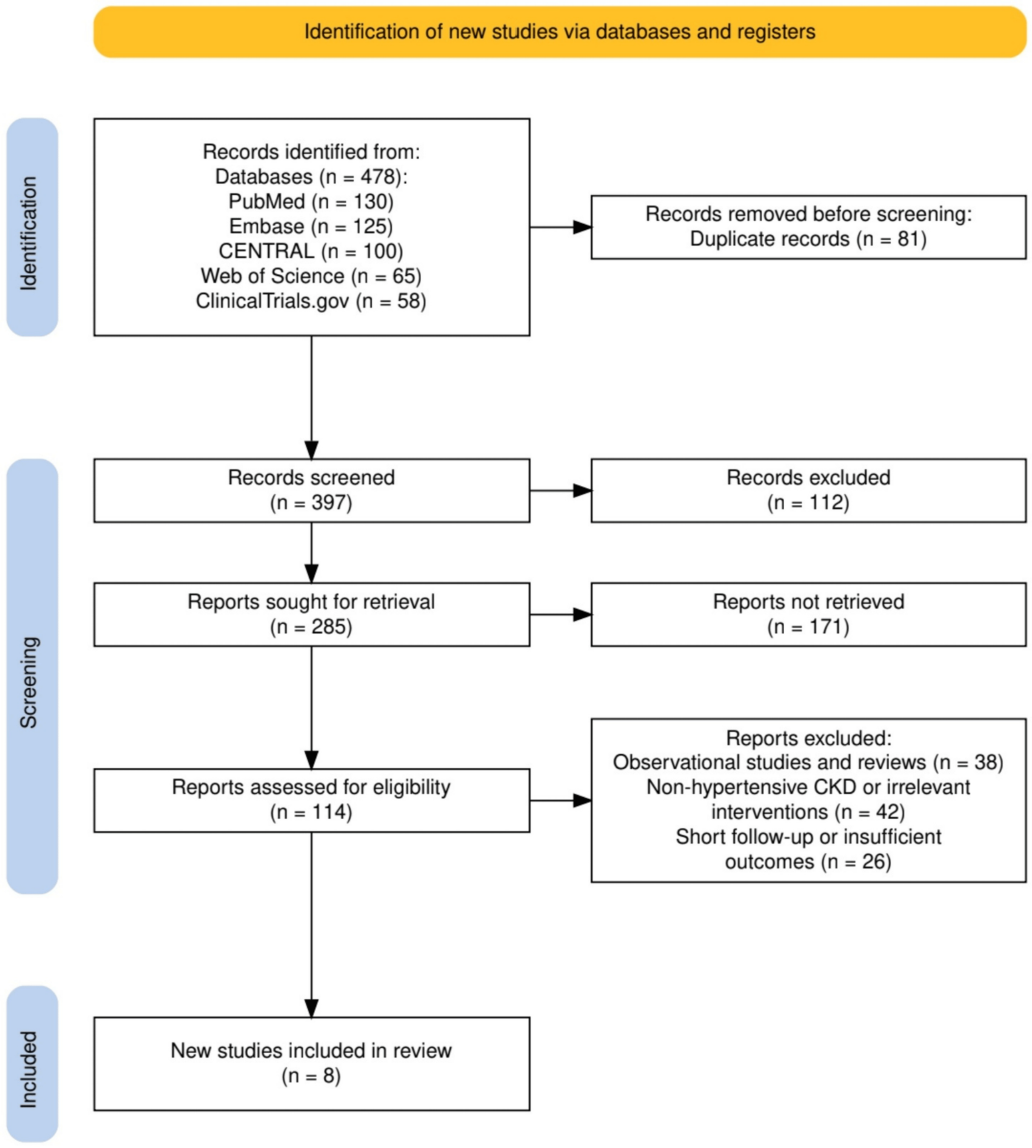
A PRISMA flowchart showcasing the study selection process PRISMA: Preferred Reporting Items for Systematic Reviews and Meta-Analyses; Embase: Excerpta Medica database; CENTRAL: Cochrane Controlled Register of Trials; CKD: chronic kidney disease

The study selection process, illustrated in Figure 1, followed a systematic approach in line with PRISMA guidelines to ensure the inclusion of high-quality and relevant studies. A total of 478 records were identified across five databases: PubMed (130), Embase (125), CENTRAL (100), Web of Science (65), and ClinicalTrials.gov (58). After removing 81 duplicate records, 397 unique studies were screened based on titles and abstracts, leading to the exclusion of 112 irrelevant records. Of the 285 reports sought for retrieval, 171 were not retrieved due to access issues or incomplete data. The remaining 114 reports underwent full-text assessment for eligibility, resulting in the exclusion of 106 studies: 38 were observational studies or reviews, 42 focused on non-hypertensive CKD populations or irrelevant interventions, and 26 had short follow-up periods or insufficient outcome reporting. Ultimately, eight studies met the inclusion criteria and were included in the final review. This rigorous selection process, as depicted in Figure 1, ensured a comprehensive and focused synthesis of the most relevant evidence.

Characteristics of the Selected Studies

The selected studies, summarized in Table 1, encompass diverse populations, including adults and children with varying stages of CKD, some with proteinuria or diabetic kidney disease (DKD), and others undergoing hemodialysis. Interventions primarily focused on ACEis, ARBs, and CCBs, with comparisons involving standard antihypertensive regimens or placebos. Key outcomes included renal parameters such as eGFR decline, proteinuria reduction, and albuminuria changes, as well as cardiovascular endpoints like blood pressure control, left ventricular mass index, and markers of inflammation. Study designs ranged from short-term trials of 16 weeks to long-term follow-ups of up to 36 months, providing a broad spectrum of evidence. However, limitations such as small sample sizes, single-center designs, and short follow-up durations in some studies may impact the generalizability of findings. This variability highlights the complexity of optimizing antihypertensive therapy in CKD and emphasizes the importance of individualized treatment approaches.

**Table 1:** Summary of the included studies in the systematic review CKD: chronic kidney disease; EGFR: estimated glomerular filtration rate; RAS: renin-angiotensin system; RAAS: renin-angiotensin-aldosterone system; ACEis: angiotensin-converting enzyme inhibitors; ARB: angiotensin receptor blockers; ESKD: end-stage kidney disease; BP: blood pressure; DKD: diabetic kidney disease; ACR: albumin-to-creatinine ratio; UACR: urinary albumin-to-creatinine ratio; SUA: serum uric acid; ADMA: asymmetrical dimethylarginine; LVMI: left ventricular mass index; hs-CRP: high-sensitivity C-reactive protein; IL-6: interleukin-6; TNF-α: tumor necrosis factor-alpha; MSBP: mean systolic blood pressure; HD: hemodialysis; CVD: cardiovascular disease

Authors (Year)	Population	Intervention	Comparison	Outcomes measured	Key findings	Limitations
Bhandari et al. (2022) [10]	411 patients with advanced CKD (eGFR <30 ml/min/1.73 m²), randomized to discontinue or continue RAS inhibitors	Discontinuation of RAS inhibitors	Continuation of RAS inhibitors	Primary: eGFR at three years; Secondary: ESKD, composite outcomes (eGFR decline >50%, renal replacement therapy), hospitalization, BP, quality of life	No significant difference in the eGFR decline rate between groups. The incidence of ESKD was slightly higher in the discontinuation group (62% vs. 56%, HR 1.28, CI 0.99–1.65).	Open-label design; potential bias in non-blinded outcome assessments; limited generalizability to broader CKD populations not meeting advanced CKD criteria.
García-Prieto et al. (2024) [11]	88 hypertensive patients aged >65 years with CKD stages 3-4 without proteinuria; mean age: 77.9±6.1 years; follow-up: 3 years	RAAS blockers (ACEi/ARB)	Standard antihypertensive treatment	Primary: eGFR decline at three years; Secondary: BP control, renal and cardiovascular events, mortality	RAAS blockers were associated with greater eGFR decline (-4.3±1.1 ml/min) compared to standard treatment (+4.6±0.4 ml/min), p=0.024. No differences in BP control, cardiovascular events, or mortality.	Small sample size; limited generalizability due to exclusion of diabetic or cardiac patients; open-label design with potential bias.
Yoo et al. (2022) [12]	341 patients with DKD; baseline urinary albumin-to-creatinine ratio (ACR): ~1376-1521 mg/gCr; follow-up: 24 weeks	Fimasartan	Losartan	Primary: Change in albuminuria (ACR); Secondary: Systolic/diastolic BP, adverse events (eGFR decline, hyperkalemia)	Fimasartan achieved significantly greater ACR reduction compared to losartan at all time points (4-24 weeks, p < 0.01). No significant differences in adverse events.	Relatively short follow-up duration for long-term CKD outcomes; specific to the DKD population, limiting generalizability to non-diabetic CKD patients.
Bryant et al. (2021) [13]	248 children with proteinuria; 47 hypertensive children randomized to losartan (23) or enalapril (24); follow-up: 36 months	Losartan	Enalapril	Changes in SUA, changes in eGFR, and proteinuria reduction	Losartan reduced SUA significantly more than enalapril (e.g., 3.69% decrease vs. 12.57% increase at 12 months, p = 0.007). SUA changes negatively correlated with eGFR changes over 36 months.	Post hoc analysis; findings may not be generalizable to adults or non-proteinuric populations; limited focus on proteinuria outcomes in broader CKD progression.
Mårup et al. (2022) [14]	140 CKD patients (eGFR 25–60 mL/min/1.73 m², UACR >500 mg/g or >200 mg/g in diabetes, plasma potassium >4.5 mmol/L); follow-up: 12 months	RAAS blockade (ACEis/ARBs + spironolactone) with patiromer	RAAS blockade (ACEis/ARBs + spironolactone) without patiromer	Primary: Change in UACR at 12 months; Secondary: CKD progression, hyperkalemia episodes, BP, eGFR, cardiovascular markers, diet, quality of life	Adding patiromer to RAAS blockade enabled intensified treatment with ACEi/ARB and spironolactone by reducing hyperkalemia, with improved albuminuria outcomes.	Open-label design with potential bias; specific to albuminuric CKD with hyperkalemia, limiting generalizability to broader CKD populations.
Ateya et al. (2022) [15]	135 hypertensive children (seven to 15 years old) on maintenance hemodialysis; 68 received ramipril, 67 placebo; follow-up: 16 weeks	Ramipril (2.5 mg once daily)	Placebo	Primary: Serum levels of asymmetrical dimethylarginine (endothelial dysfunction) and hs-CRP (inflammation); Secondary: IL-6, TNF-α, BP reduction, safety parameters (e.g., hyperkalemia)	Ramipril significantly reduced markers of endothelial dysfunction (-79.6%) and inflammation (hs-CRP -46.5%, IL-6 -27.1%, TNF-α -51.7%, all p < 0.001). Ramipril lowered systolic and diastolic BP significantly more than placebo. No severe hyperkalemia or serious adverse events were reported.	Short follow-up period; pediatric and dialysis population may limit generalizability to adults or non-dialysis CKD patients.
Youssef et al. (2021) [16]	46 hypertensive patients on maintenance HD with no history of CVD; follow-up: six months	Amlodipine (10 mg/day)	Bisoprolol (10 mg/day)	Changes in LVMI and ADMA; BP control	Amlodipine significantly reduced LVMI (35 ± 34.2 gm/m² vs. 9.8 ± 35.9 gm/m², P = .017) and ADMA levels (P = .001). Both groups achieved similar BP reductions.	Small sample size; single-center study; short follow-up period limits evaluation of long-term cardiovascular and renal outcomes.
Jankauskiene et al. (2021) [17]	127 children aged between one to five years with hypertension (63 with CKD); randomized to valsartan 0.25 mg/kg/day or four mg/kg/day; follow-up: six weeks double-blind phase, 20-week open-label phase	Valsartan (4 mg/kg/day)	Valsartan (0.25 mg/kg/day)	Primary: Reduction in MSBP; Secondary: Safety (adverse events, serum potassium changes)	Valsartan 4 mg/kg/day significantly reduced MSBP compared to 0.25 mg/kg/day in the overall group (-8.5 vs. -4.1 mmHg, p = .0157) and in the CKD subgroup (-9.2 vs. -1.2 mmHg, p = .0096). Adverse events were less frequent with 4 mg/kg/day.	The pediatric population may not generalize to adults; short follow-up for evaluating long-term CKD outcomes; and higher serum potassium increases in CKD patients were noted.

Quality Assessment

The quality assessment of the included studies, presented in Table 2, highlights the overall methodological rigor while identifying areas of potential bias. Most studies demonstrated low risk across key domains, including randomization processes, adherence to interventions, outcome measurement, and reporting of results. Randomization was clearly described in nearly all trials, with several studies employing double-blind designs to minimize deviations from intended interventions. Outcome measures, such as eGFR, proteinuria, and blood pressure, were assessed using standardized methods, ensuring reliability. However, some studies raised concerns, particularly regarding small sample sizes and post hoc analyses, which could introduce bias. For instance, concerns were noted in single-center trials or studies with retrospective components, such as those by Bryant et al. [13] and Youssef et al. [16], where randomization processes and missing data presented limitations. Despite these issues, the overall risk of bias was deemed low for most studies, reinforcing the reliability of the evidence synthesized in this review.

**Table 2:** The quality assessment of the included studies BP: blood pressure; LVMI: left ventricular mass index; ADMA: asymmetrical dimethylarginine; MSBP: mean systolic blood pressure

Study	Randomization process	Deviations from intended interventions	Missing outcome data	Measurement of the outcome	Selection of the reported result	Overall risk of bias
Bhandari et al. (2022) [10]	Low risk: Randomized and clearly described	Low risk: Adherence was monitored	Low risk: Minimal missing data	Low risk: Outcomes measured appropriately	Low risk: Outcomes fully reported	Low risk
García-Prieto et al. (2024) [11]	Low risk: Proper randomization described	Low risk: No deviations noted	Some concerns: Small sample size	Low risk: Standardized measurements	Low risk: No evidence of selective reporting	Low risk
Yoo et al. (2022) [12]	Low risk: Randomization process well documented	Low risk: Double-blind study design ensured adherence	Low risk: Minimal missing data	Low risk: Albuminuria and BP measured appropriately	Low risk: Full reporting of outcomes	Low risk
Bryant et al. (2021) [13]	Some concerns: Post hoc analysis	Some concerns: Retrospective approach	Some concerns: Missing data possible	Low risk: Standardized outcome measures	Low risk: No evidence of selective reporting	Some concerns
Mårup et al. (2022) [14]	Low risk: Randomized, open-label trial	Low risk: Adherence monitored effectively	Low risk: Minimal missing data	Low risk: Primary and secondary outcomes clearly defined	Low risk: Outcomes fully reported	Low risk
Ateya et al. (2022) [15]	Low risk: Proper randomization	Low risk: Double-blind design	Low risk: Minimal missing data	Low risk: Biomarkers and BP measured appropriately	Low risk: Full outcome reporting	Low risk
Youssef et al. (2021) [16]	Some concerns: Single-center trial, randomization process unclear	Low risk: Deviations unlikely	Some concerns: Small sample size	Low risk: LVMI and ADMA measured using standard methods	Low risk: No selective reporting	Some concerns
Jankauskiene et al. (2021) [17]	Low risk: Randomized, double-blind study	Low risk: Adherence monitored effectively	Low risk: Minimal missing data	Low risk: MSBP measured using standardized methods	Low risk: No evidence of selective reporting	Low risk

Discussion

This systematic review provides a comprehensive evaluation of the comparative efficacy and safety of antihypertensive therapies, including ACEis, ARBs, and CCBs, in patients with CKD. Across the included studies, ACEis and ARBs consistently demonstrated efficacy in reducing proteinuria and slowing CKD progression, particularly in patients with albuminuria. For instance, Bhandari et al. [10] found no significant differences in eGFR decline between continuation and discontinuation of RAAS inhibitors, although discontinuation slightly increased the risk of ESKD. Similarly, Yoo et al. [12] showed that fimasartan, an ARB, outperformed losartan in reducing albuminuria in DKD, highlighting the potential for ARBs to offer superior renal protection in specific populations. The study by Bryant et al. [13] further emphasized the unique benefits of losartan in reducing serum uric acid (SUA), which negatively correlated with eGFR changes over time, suggesting an additional renoprotective mechanism in children with proteinuria.

Calcium channel blockers also showed promise, particularly in managing blood pressure and cardiovascular outcomes. Youssef et al. [16] demonstrated that amlodipine significantly reduced left ventricular mass index (LVMI) and asymmetrical dimethylarginine (ADMA) levels in hypertensive patients on hemodialysis, suggesting cardiovascular benefits beyond blood pressure control. In pediatric patients, Jankauskiene et al. [17] found that higher doses of valsartan (an ARB) resulted in significantly greater reductions in mean systolic blood pressure (MSBP) compared to lower doses, with fewer adverse events observed in the higher dose group. The inclusion of adjunctive therapies, such as patiromer, as shown by Mårup et al. [14], enabled intensified RAAS blockade by mitigating hyperkalemia, leading to improved albuminuria outcomes. Collectively, these findings underscore the importance of tailoring antihypertensive therapy to patient-specific characteristics, such as proteinuria presence, CKD stage, and comorbid conditions, to optimize renal and cardiovascular outcomes.

The findings of this systematic review align with existing literature emphasizing the efficacy of ACEis and ARBs in managing hypertension among CKD patients [18]. Prior studies have demonstrated that ACEis and ARBs not only effectively reduce blood pressure but also slow CKD progression and decrease proteinuria. For instance, a meta-analysis highlighted the superiority of ACEis over ARBs in preventing kidney failure and reducing all-cause mortality in CKD patients [19]. Additionally, research comparing CCBs to ACEis and ARBs in hypertensive CKD patients found that while all three classes effectively lower blood pressure, ACEis and ARBs offer added renal protection by reducing proteinuria and slowing disease progression [3].

However, some discrepancies exist between our findings and certain studies, particularly concerning the use of renin-angiotensin system (RAS) inhibitors in advanced CKD stages. While our review suggests the potential benefits of continuing RAS inhibitors in advanced CKD, other studies have raised concerns about their safety and efficacy in such populations. For example, a recent analysis questioned the initiation of ACEi or ARBs in patients with advanced CKD due to potential risks, including hyperkalemia and acute kidney injury [20]. These discrepancies may stem from differences in study populations, methodologies, and outcome measures. Variations in patient demographics, CKD stages, and concurrent comorbidities across studies could contribute to differing results. Additionally, methodological differences, such as study design (e.g., randomized controlled trials vs. observational studies), sample sizes, and follow-up durations, may influence outcomes. Furthermore, the specific outcomes measured, such as eGFR decline, proteinuria reduction, or cardiovascular events, can lead to varying interpretations of the efficacy and safety of these interventions.

The findings of this systematic review have significant clinical implications for the management of hypertensive patients with CKD. Both ACEis and ARBs demonstrated strong renal protective effects, particularly in reducing proteinuria and slowing CKD progression, making them first-line choices for patients with proteinuric CKD [21]. Angiotensin receptor blockers, such as fimasartan and losartan, offer viable alternatives for those who cannot tolerate ACEis due to adverse effects like cough or angioedema. Calcium channel blockers, while less effective in reducing proteinuria, are beneficial for patients with resistant hypertension or in cases where RAAS blockade is contraindicated [22]. Additionally, the inclusion of adjunctive therapies, like potassium binders, may enable intensified RAAS blockade while mitigating hyperkalemia, as seen in patients with albuminuric CKD and elevated potassium levels. These findings underscore the importance of individualized therapy based on patient-specific factors, such as the presence of proteinuria, cardiovascular risk, CKD stage, and tolerance to medication, to optimize both renal and cardiovascular outcomes in this high-risk population [23].

This systematic review is strengthened by its methodological rigor, including adherence to PRISMA guidelines, a comprehensive and systematic search strategy, and well-defined inclusion and exclusion criteria. By focusing on recent RCTs published within the last five years, the review provides an up-to-date synthesis of evidence comparing ACEis, ARBs, and CCBs in hypertensive CKD patients. Additionally, the review fills an important gap in the literature by addressing the comparative efficacy of these therapies across diverse CKD populations, including those with and without proteinuria, and evaluating their impact on both renal and cardiovascular outcomes. These contributions are valuable for refining clinical guidelines and informing evidence-based treatment strategies.

However, this review has some limitations. The included studies varied in sample size, with several having small populations that may limit the generalizability of their findings. Additionally, the short follow-up periods in many trials hinder the ability to assess long-term outcomes, such as progression to ESRD or long-term cardiovascular events. Methodological concerns, such as open-label designs and potential biases in some studies, may also affect the reliability of certain findings. From a review process perspective, the exclusion of non-English studies and reliance on published data may introduce selection bias and limit the comprehensiveness of the synthesis. Despite these limitations, the review provides critical insights into optimizing antihypertensive therapy for CKD patients.

Future research should focus on addressing the gaps and limitations identified in this review, particularly through larger, multicenter trials with extended follow-up durations to evaluate long-term renal and cardiovascular outcomes in hypertensive CKD patients. Studies should aim to better understand the comparative efficacy of ACEis, ARBs, and CCBs in specific subpopulations, such as those with advanced CKD or non-proteinuric CKD, to tailor therapeutic recommendations more effectively. Emerging areas, such as the role of combination therapies, including the use of potassium binders to enhance RAAS blockade, warrant further exploration to optimize treatment in patients prone to hyperkalemia. Additionally, future research should investigate the integration of novel biomarkers for CKD progression and cardiovascular risk to refine therapeutic monitoring and predict patient outcomes [24]. As the evidence base grows, these studies will be instrumental in shaping more comprehensive and individualized treatment strategies for this high-risk population.

## Conclusions

This systematic review underscores the critical role of ACEIs and ARBs as cornerstone therapies in managing hypertension among CKD patients, particularly for those with proteinuria, due to their demonstrated ability to reduce proteinuria and slow CKD progression. Calcium channel blockers, while less effective in renal protection, provide valuable options for patients with resistant hypertension or contraindications to RAAS inhibitors. The findings highlight the importance of individualized treatment strategies that consider patient-specific factors such as CKD stage, proteinuria presence, cardiovascular risk, and medication tolerance. By synthesizing recent high-quality evidence, this review contributes to refining clinical guidelines and addressing gaps in therapeutic optimization for hypertensive CKD populations. Moving forward, further research into combination therapies and long-term outcomes is essential to enhance treatment efficacy and improve patient outcomes. The key takeaway is the necessity of personalized, evidence-based approaches to manage hypertension effectively and mitigate CKD progression and its associated risks.
